# Milestones in understanding transport, sensing, and signaling of the plant nutrient phosphorus

**DOI:** 10.1093/plcell/koad326

**Published:** 2024-01-02

**Authors:** Shu-Yi Yang, Wei-Yi Lin, Yi-Min Hsiao, Tzyy-Jen Chiou

**Affiliations:** Institute of Plant Biology, National Taiwan University, Taipei 106319, Taiwan; Department of Agronomy, National Taiwan University, Taipei 106319, Taiwan; Agricultural Biotechnology Research Center, Academia Sinica, Taipei 115201, Taiwan; Agricultural Biotechnology Research Center, Academia Sinica, Taipei 115201, Taiwan

## Abstract

As an essential nutrient element, phosphorus (P) is primarily acquired and translocated as inorganic phosphate (Pi) by plant roots. Pi is often sequestered in the soil and becomes limited for plant growth. Plants have developed a sophisticated array of adaptive responses, termed P starvation responses, to cope with P deficiency by improving its external acquisition and internal utilization. Over the past 2 to 3 decades, remarkable progress has been made toward understanding how plants sense and respond to changing environmental P. This review provides an overview of the molecular mechanisms that regulate or coordinate P starvation responses, emphasizing P transport, sensing, and signaling. We present the major players and regulators responsible for Pi uptake and translocation. We then introduce how P is perceived at the root tip, how systemic P signaling is operated, and the mechanisms by which the intracellular P status is sensed and conveyed. Additionally, the recent exciting findings about the influence of P on plant-microbe interactions are highlighted. Finally, the challenges and prospects concerning the interplay between P and other nutrients and strategies to enhance P utilization efficiency are discussed. Insights obtained from this knowledge may guide future research endeavors in sustainable agriculture.

## Teaser

This article reviews the molecular mechanisms of phosphorus transport, sensing and signaling; and highlights recent findings about the impact of phosphorus on plant-microbe interactions.

## Introduction

Phosphorus (P) is a macronutrient that is indispensable for growth, development, and reproduction because it is an essential component of several important biomolecules, such as DNA, RNA, ATP, NADPH, and phospholipids, and is also involved in respiration and photosynthesis (Raghothama 1999; Jiang *et al*. 2019a). Orthophosphate (Pi, H_2_PO_4_^−^/HPO_4_^2−^) is the predominant form of P absorbed by plant roots through loading into the xylem and translocation into shoots. Still, it is quickly fixed by Fe^3+^ and Al^3+^ at low pH (<5 or acidic soils) or by tricalcium at high pH (>7 or alkaline soils), resulting in low availability and mobility in the soil (Holford 1997; Prathap *et al*. 2022). Modern agriculture leans heavily on P fertilizer to cope with the low available soil Pi. However, due to the low efficiency of applied P absorbed by plants and the finite nature of P fertilizer, which is resourced from natural P rock reserves, increasing the P use efficiency (PUE) of crops has become an important issue to fulfill the demand for food security and agricultural sustainability (Baker *et al*. 2015; van de Wiel *et al*. 2016; Heuer *et al*. 2017). Endeavors to increase PUE will rely on knowledge of how plants sense and respond to environmental P levels.

**Figure 1.**
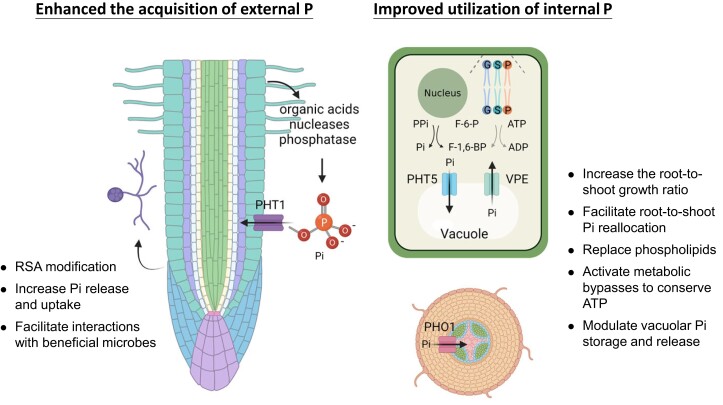
An overview of adaptive P starvation responses in plants. There are 2 major categories of adaptive PSRs. One is to enhance the acquisition of external P, including root system architecture (RSA) modification; increased Pi release via acid/enzyme secretion; elevated Pi uptake through PHT1 transporters; and facilitated interactions with beneficial microbes such as arbuscular mycorrhizal fungi. The other is to improve utilization of internal P, including increased root-to-shoot growth ratio, PHO1-mediated root-to-shoot Pi reallocation, phospholipid replacement with galacto- and sulfolipids, ATP-conserving metabolic bypasses, and regulation of vacuolar Pi storage/release by PHT5 and VPE. Created with BioRender.com.

In response to P deficiency, plants have developed sophisticated mechanisms to enhance the external acquisition and improve the internal utilization of P (Fig. 1), designated as P starvation responses (PSRs). Local PSRs are regulated by the external P concentration in the soil surrounding the root and are responsible for enhancing the acquisition of external P. Systemic PSRs depend on the adjustment of internal P concentration and improve the utilization of internal P through P recycling and reallocation via coordinating with external P availability (Bates and Lynch 1996; Thibaud *et al*. 2010; Lin *et al*. 2014; Chien *et al*. 2018). Regarding the enhanced acquisition of external P, the modification of the root system architecture, including an increase in the number of lateral roots and root hairs and a reduction of the primary root growth, allows plants to forage for P in the topsoil and enlarge root-soil surface efficiently (Bates and Lynch 2001; López-Bucio *et al*. 2003; Lynch 2011). In addition, increased high-affinity Pi transporter (PHT1) activity in the root surface facilitates Pi uptake. The organic acids, nucleases, and phosphatase secreted from the root enable Pi release from insoluble or organic matter (Taylor *et al*. 1993; Bariola *et al*. 1994; Raghothama 1999; Plaxton and Tran 2011). Interactions with beneficial microorganisms triggered by P starvation could also promote P acquisition (Oldroyd and Leyser 2020). Regarding the improved utilization of internal P, an increase in the root-to-shoot growth ratio, a reduction in shoot branching, and an increase in the erectness of leaves could allow plants to adjust growth and photosynthetic efficiency to adapt to P deficiency (Ruan *et al*. 2018; López-Bucio *et al*. 2003). The increase in the xylem loading activity of Pi facilitates root-to-shoot Pi reallocation (Hamburger *et al*. 2002). The replacement of phospholipids by galacto- and sulfolipids, the activation of metabolic bypasses to conserve ATP, and the maintenance of cytosolic Pi homeostasis by modulating vacuolar Pi storage and release also occur (Härtel *et al*. 2001; Kelly and Dörmann 2002; Gaude *et al*. 2008; Plaxton and Tran 2011; Wang *et al*. 2012; Nakamura 2013; Okazaki *et al*. 2013; Liu *et al*. 2015, 2016; Wang *et al*. 2015; Xu *et al*. 2019).

**Figure 2.**
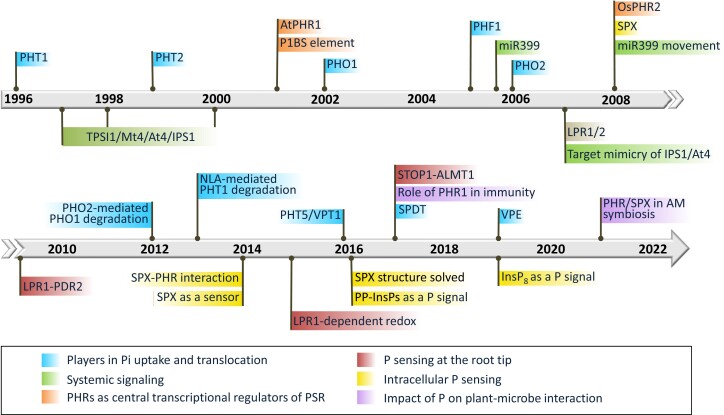
Milestones in understanding the genes involved in P transport, sensing, and signaling pathways. The genes with different roles in Pi uptake and translocation; local (root tip), systemic, and intracellular P sensing and signaling; and P-mediated plant-microbe interactions are depicted along the timeline and chronologically arranged according to the original publication dates.

In this review, we first summarize the historical key discoveries pertaining to the molecular players involved in P sensing, signaling, uptake, and utilization in plants since PHT1 Pi transporters were identified in 1996 (Fig. 2). We then highlight the major players and regulators in Pi uptake and translocation in coordinating PSRs. We illustrate local P sensing at the root tip, systemic P signaling, and intracellular P sensing. Furthermore, the recent exciting findings about P in plant-microbe interactions are discussed. Finally, the remaining challenges and perspectives on crosstalk with other nutrients and how to improve PUE are presented.

## Historical overview—the key players in P transport, sensing, and signaling

This section briefly introduces the discoveries of key players in Pi transport, sensing, and signaling chronologically (Fig. 2). The details are elaborated afterward.

Since the identification of the genes encoding Pi transporters, molecular studies on P acquisition and its regulation have been inspired and enthusiastically pursued. Members of the plasma membrane–localized Pi transporter PHT1 family were first identified from Arabidopsis by yeast functional complementation in 1996 (Muchhal *et al*. 1996), followed by the second Pi transporter PHT2 in 1999, which is localized in the chloroplast (Daram *et al*. 1999). Later, in 2002, PHOSPHATE 1 (PHO1) was identified as a Pi efflux transporter mediating root-to-shoot Pi translocation (Hamburger *et al*. 2002). Then, in 2017, an additional group of Pi transporters belonging to the family of sulfate transporters (SULTRs), named SULTR-like phosphorus distribution transporter (SPDT), was uncovered (Yamaji *et al*. 2017). More recently, Pi transporters facilitating Pi translocation between the cytoplasm and vacuole have been identified, including Arabidopsis PHT5;1/VPT1 (VACUOLAR PHOSPHATE TRANSPORTER), a Pi influx transporter; and rice VPE1 and VPE2 (VACUOLAR PHOSPHATE EFFLUX), Pi efflux transporters (Liu *et al*. 2015, 2016; Xu *et al*. 2019). After uncovering the molecular identity of these Pi transporters, their regulation at the transcriptional and posttranslational levels was revealed. For example, upregulation of PHT1 transcription by low P is mediated by PHOSPHATE STARVATION RESPONSE (PHR) proteins binding to PHR1 binding sequence (P1BS, GNATATNC) (Rubio *et al*. 2001; Zhou *et al*. 2008), the proper targeting of PHT1 Pi transporters to plasma membranes requires PHOSPHATE TRANSPORTER TRAFFIC FACILITATOR 1 (PHF1) (Gonzalez *et al*. 2005), and the protein abundance of PHT1 and PHO1 in response to Pi availability is controlled by PHOSPHATE 2 (PHO2/UBC24), a ubiquitin E2 conjugase, (Aung *et al*. 2006; Bari *et al*. 2006; Liu *et al*. 2012; Huang *et al*. 2013), and/or NITROGEN LIMITATION ADAPTATION (NLA), a RING-type E3 ligase (Lin *et al*. 2013). It is worth mentioning that PHO1 and PHO2 were identified initially from genetic screenings through having significantly reduced or increased shoot Pi content, respectively (Poirier *et al*. 1991; Delhaize and Randall 1995).

To coordinate the P acquisition in the roots and P utilization at the whole-plant level for optimizing plant growth and development, sensing and signaling P availability are requisite. P sensing and signaling can be classified into local and systemic. The local signaling to sense external P at the root tips was shown to be mediated by Low Phosphate Root1 (LPR1) and LPR2, 2 multicopper oxidases (Svistoonoff *et al*. 2007). Other components, such as PHOSPHATE DEFICIENCY RESPONSES 2 (PDR2) and ALUMINUM ACTIVATED MALATE TRANSPORTER 1 (ALMT1), were later found to work together with LPR1 to regulate root meristem differentiation (Ticconi *et al*. 2009; Balzergue *et al*. 2017). On the other hand, systemic signaling plays an essential role in the improved utilization of internal P. From 1997 to 2000, noncoding RNAs (*TPSI1*, *Mt4*, *At4* and *IPS1*) were discovered in different plant species that are highly induced by P starvation and share a conserved 22-nucleotide sequence (Liu *et al*. 1997; Burleigh and Harrison 1999; Martín *et al*. 2000; Shin *et al*. 2006). Their biological function remained unknown until the discovery of microRNA399 (miR399), the first miRNA identified to be involved in P starvation response (Fujii *et al*. 2005). Upon P starvation, miR399 is upregulated, which suppresses *PHO2* expression and leads to the activation of Pi uptake and translocation (Aung *et al*. 2006; Bari *et al*. 2006; Chiou *et al*. 2006). *AT4*/*IPS1* homologs function to modulate the action of miR399 by sequestering miR399 through the 22 conserved nucleotides via a mechanism of target mimicry (Franco-Zorrilla *et al*. 2007). Interestingly, miR399 was found to serve as a shoot-to-root systemic signal (Lin *et al*. 2008; Pant *et al*. 2008).

With regard to intracellular Pi sensing and signaling, SPX proteins were discovered to be involved in regulating PSR genes in 2008 (Duan *et al*. 2008), and their role as P sensors through SPX-PHR interaction was reported in 2014 (Puga *et al*. 2014; Wang *et al*. 2014b). SPX proteins function as repressors to sequester PHRs from transcriptional activation of PSRs. Notably, a structure-function analysis revealed that inositol polyphosphate (InsP) and inositol pyrophosphate (PP-InsP) rather than Pi could be bound by SPX in vitro, but PP-InsPs could potentially serve as the pertinent signaling molecules in vivo (Wild *et al*. 2016). Later genetic analyses revealed that bis-diphosphoinositol tetrakisphosphate 1,5(PP)_2_-InsP_4_ (1,5-InsP_8_) acts as a bona fide intracellular signaling molecule to regulate PSRs in plants (Dong *et al*. 2019a; Zhu *et al*. 2019).

Plants actively modulate the interactions with various microbes to adapt to the Pi availability. Strikingly, PHR was discovered to serve as a negative and positive regulator to mediate immune-related genes and arbuscular mycorrhizal-related genes, respectively (Castrillo *et al*. 2017; Isidra-Arellano *et al*. 2021; Shi *et al*. 2021; Das *et al*. 2022). The role of SPX in arbuscular mycorrhizal (AM) symbiosis was also revealed (Shi *et al*. 2021; Wang *et al*. 2021; Liao *et al*. 2022), suggesting the central role of SPX-PHR in integrating signals involved in nutritional response and biotic interaction.

## Players in Pi uptake and translocation

**Figure 3.**
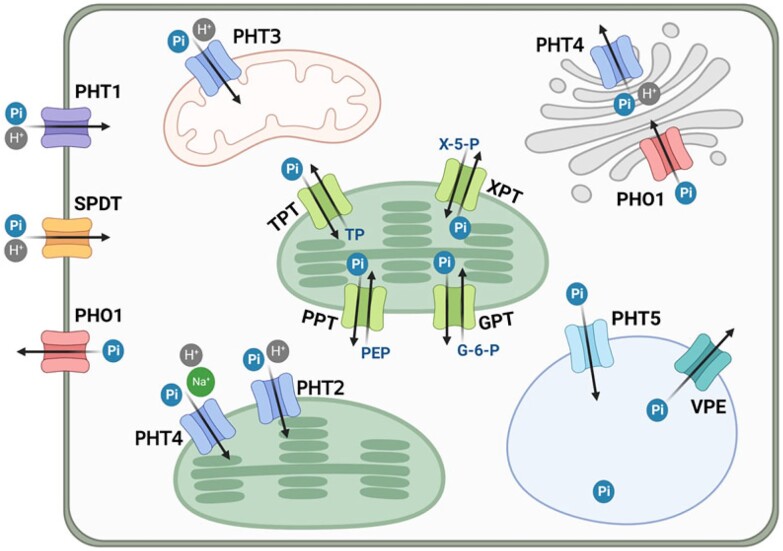
Subcellular localization of players in Pi uptake and translocation. Arrows indicate the direction of Pi transport. Plasma membrane-localized PHT1 and SPDT are responsible for Pi uptake. PHT2 (chloroplast), PHT3 (mitochondria), and PHT4 (the Golgi apparatus, chloroplast, and nonphotosynthetic plastid) mediate Pi import or export from the corresponding organelles. Pi influx and efflux through the tonoplast are operated by PHT5 and VPE, respectively. PHO1 protein localizes to the plasma membrane and Golgi bearing the Pi efflux activity for Pi allocation. Pi translocators (PT) localized in the inner envelope membrane of plastids transport Pi in exchange with different substrates, including triose-phosphate/phosphate translocator (TPT), phosphoenolpyruvate/phosphate translocator (PPT), glucose-6-phosphate/phosphate translocator (GPT), and pentose xylulose-5-phosphate/phosphate translocator (XPT). Coupling with proton (H^+^) or sodium (Na^+^) transport is indicated. Created with BioRender.com.

Pi is initially acquired by the root epidermal and/or cortical cells and subsequently transported radially to the central vascular tissues where Pi is loaded into the xylem for translocation up to the shoot. Pi is then allocated among different tissues and distributed in organelles to sustain growth and development or stored inside vacuoles when excessive. Therefore, Pi transporters in the plasma membrane or organellar membranes are essential to facilitate Pi transport across membranes for uptake, distribution and remobilization (Fig. 3). While vegetative cells generally store Pi inside the vacuole, the primary form of P stored in the vacuole of seeds is inositol hexakisphosphate (InsP_6_, phytate) mediated by MULTIDRUG RESISTANCE-ASSOCIATED PROTEIN 5 (MRP5), a vacuolar InsP_6_ transporter (Nagy *et al*. 2009).

Pi transporters comprise different families, such as the phosphate transporter (PHT) family, the SYG1/Pho81/XPR1 (SPX) domain-containing protein family and the SPDT family. Members in the PHT family are classified into 5 types according to sequence identity and subcellular localization (Versaw and Garcia 2017), comprising PHT1 (plasma membrane), PHT2 (chloroplast), PHT3 (mitochondria), PHT4 (the Golgi apparatus, chloroplast and non-photosynthetic plastid) and PHT5 (vacuole). SPX domain-containing proteins are divided into 4 subfamilies: SPX, SPX-EXS (ERD1/XPR1/SYG1), SPX-MFS (Major Facilitator Superfamily), and SPX-RING (Really Interesting New Gene). Among them, SPX-MFS domain- and SPX-EXS domain-proteins have been identified as Pi transporters (e.g. members of the PHT5 family and PHO1, respectively) (Wang *et al*. 2012, 2015; Liu *et al*. 2015, 2016).

### PHT1 transporters

PHT1 transporters are proton-Pi symporters and play a critical role in Pi acquisition from the soil and in Pi translocation between tissues (Nussaume *et al*. 2011; Remy *et al*. 2012; Lopez-Arredondo *et al*. 2014; Ayadi *et al*. 2015). There are 13 members in rice (*Oryza sativa*) and 9 in *Arabidopsis thaliana*. Most of them are expressed in roots and upregulated by the PHR transcription factor under P starvation (Karthikeyan *et al*. 2002; Mudge *et al*. 2002). In Arabidopsis, Pi uptake is mainly mediated by PHT1;1 and PHT1;4 (Shin *et al*. 2004; Remy *et al*. 2012; Ayadi *et al*. 2015), and root-to-shoot mobilization is facilitated by PHT1;8/PHT1;9 (Lapis-Gaza *et al*. 2014). In rice, OsPT2, a low-affinity Pi transporter exclusively expressed in the stele, facilitates the movement of Pi from roots to shoots. In contrast, OsPT6 and OsPT8 function as high-affinity Pi transporters responsible for Pi uptake and subsequent translocation (Ai *et al*. 2009; Jia *et al*. 2011). OsPT9 and OsPT10, 2 other high-affinity Pi transporters, are also involved in Pi uptake (Wang *et al*. 2014a). The expression of these 5 rice PHT1 transporters were all induced by Pi starvation in the roots (Ai *et al*. 2009; Jia *et al*. 2011; Wang *et al*. 2014a). The members of *PHT1* are also expressed in the reproductive tissues responsible for anther and embryo development, for example, Arabidopsis *PHT1;6* and *PHT1;7* and rice *OsPht1;7* in the anthers (Mudge *et al*. 2002; Dai *et al*. 2022) and *OsPht1;4* in the embryo (Zhang *et al*. 2015).

Regarding the proper localization of PHT1 proteins, PHOSPHATE TRANSPORTER TRAFFIC FACILITATOR 1 (PHF1), related to yeast SECRETORY 12 (SEC12), facilitates the exit of PHT1 proteins from the endoplasmic reticulum (ER) to plasma membranes in rice and Arabidopsis (Gonzalez *et al*. 2005; Bayle *et al*. 2011; Chen *et al*. 2011b). Additionally, the phosphorylation of rice OsPHT1;8 at Ser-517 by Casein Kinase 2 (OsCK2) interfered with the interaction between OsPHT1;8 and PHF1 and resulted in ER retention of OsPHT1;8 (Chen *et al*. 2015). Intriguingly, PHT1 protein abundance in the ER is controlled by PHO2-mediated degradation according to cellular P status, which determines the PHT1 amount directed to the plasma membrane (Huang *et al*. 2013).

Once at plasma membranes, PHT1 proteins are regulated by NLA-mediated degradation, a RING-type ubiquitin E3 ligase containing the SPX domain that can perceive cellular P levels (as discussed below) (Lin *et al*. 2013; Park *et al*. 2014; Yue *et al*. 2017; Yang *et al*. 2020). It was proposed that the cooperation between NLA and PHO2 controls the final protein level of PHT1 (Lin *et al*. 2013; Park *et al*. 2014). Intriguingly, different from Arabidopsis AtNLA, which is cleaved by P starvation-induced miR827 (Hsieh *et al*. 2009), rice *OsNLA1* is not the target of miR827, but its upstream open reading frame (uORF) is essential for its P-induced transcriptional expression (Lin *et al*. 2018; Yang *et al*. 2020).

### PHT2/3/4 transporters

PHT2, PHT3, and PHT4 are localized to organelles, such as chloroplasts/plastids, mitochondria, and the Golgi apparatus. In Arabidopsis, PHT4;1, PHT4;2, and PHT2;1 control the Pi concentration in chloroplasts and regulate ATP synthesis and starch accumulation (Versaw and Harrison 2002; Karlsson *et al*. 2015; Miyaji *et al*. 2015). Similarly, mitochondrial localized PHT3 also plays a role in ATP metabolism (Zhu *et al*. 2012). Unexpectedly, PHT4;4 was shown to transport ascorbate into chloroplasts required to tolerate high light stress (Miyaji *et al*. 2015). In rice, OsPHT2;1 is also characterized as a chloroplast Pi influx transporter. Overexpression and loss of OsPHT2;1 result in altered Pi content in the shoot under low P conditions (Liu *et al*. 2020). How different organelles coordinate with each other to mediate cellular Pi homeostasis between the cytosol and organelles awaits more studies.

### Vacuolar Pi transporters—PHT5 and VPE

Inside plant vegetative cells, the vacuole is the largest organelle and stores about 70% to 95% of the intracellular Pi (Yang *et al*. 2017), thus serving as an important reservoir in buffering cytoplasmic Pi concentrations. The Pi influx into the vacuole is operated by Arabidopsis PHT5 or VPT and rice SPX-MFS (Wang *et al*. 2012; Liu *et al*. 2015, 2016). These transporters contain N-terminal SPX and C-terminal MFS domains. OsSPX-MFS1 controls Pi homeostasis in leaves and mediates Pi influx to yeast vacuoles when heterologously expressed (Wang *et al*. 2012; Liu *et al*. 2016). Loss of Arabidopsis PHT5;1/VPT1 reduces total Pi level but results in necrotic leaves during P replenishment after starvation (Liu *et al*. 2015, 2016). Patch clamp analysis revealed a reduced Pi influx current in the vacuoles of *vpt1* mutants compared with wild-type ones (Liu *et al*. 2015). In addition, decreased vacuolar Pi and increased cytosolic Pi levels were observed in *pht5* mutants by ^31^P-NMR analysis or FRET-based Pi sensor (Liu *et al*. 2016; Sahu *et al*. 2020). Notably, the expression of *AtPHT1s* is affected in *pht5* mutants and *PHT5* overexpressor, implying that coordination between the storage capacity and acquisition activity would be achieved to maintain cytosolic Pi concentration (Liu *et al*. 2016). Conversely, rice vacuolar Pi efflux proteins VPE1/2 operate Pi efflux from the vacuole. The *vpe1/2* double mutant and overexpression plants show higher and lower vacuolar Pi levels, respectively (Xu *et al*. 2019). Unlike *VPE1/2*, the expression of *PHT5* is not responsive to P status, suggesting that the activity of PHT5 might be regulated at the protein level (Liu *et al*. 2016; Xu *et al*. 2019). Indeed, a recent study showed that the activity of PHT5 is impaired under Pi-deficient conditions or when the InsP/PP-InsP binding pocket in its SPX domain is mutated, thus linking the cellular P status to modulate the vacuolar Pi storage activity (Luan *et al*. 2022).

### PHO1

Arabidopsis PHO1 and its close homolog PHO1;H1 with Pi efflux activity were able to facilitate the unloading of Pi into the xylem apoplastic space and allow for the subsequent translocation of Pi to the shoots (Poirier *et al*. 1991; Hamburger *et al*. 2002; Arpat *et al*. 2012; Liu *et al*. 2012). The significance of PHO1 in the root-to-shoot Pi allocation is supported by the higher Pi accumulation in roots and reduced Pi levels in shoots of *pho1* mutants in Arabidopsis and rice (Poirier *et al*. 1991; Secco *et al*. 2010; Che *et al*. 2020). AtPHO1 also facilitates the transfer of Pi from maternal tissues to developing seeds in the chalazal seed coat (Vogiatzaki *et al*. 2017). It is also expressed in leaf guard cells to play a role in the stomatal response to abscisic acid (ABA) (Zimmerli *et al*. 2012). In rice, OsPHO1;2 are involved in the allocation of Pi during grain filling (Che *et al*. 2020; Ma *et al*. 2021). In *Medicago truncatula*, PHO1 mediates the transfer of Pi from nodule-infected cells to bacteroids (Nguyen *et al*. 2021). Earlier results showed that the subcellular localization of Arabidopsis PHO1 is in endomembrane systems, such as the Golgi and trans-Golgi networks, different from the plasma membrane localization of OsPHO1;2 (Arpat *et al*. 2012; Liu *et al*. 2012; Che *et al*. 2020). Nevertheless, a recent report showed that AtPHO1 is constitutively internalized from the plasma membrane (Vetal and Poirier 2023).

Arabidopsis PHO1 is negatively regulated at the translational level by its uORF (Reis *et al*. 2020) or at the transcriptional level by WRKY6 (Chen *et al*. 2009). In rice, the OsPHO1;2 mRNA translation can be enhanced by interacting with its cis-natural antisense transcript (Jabnoune *et al*. 2013; Reis *et al*. 2021). PHO1 has a cytosolic N-terminal SPX domain, 4 transmembrane domains, and a C-terminal EXS domain (Wege *et al*. 2016). The EXS domain is required for PHO1 membrane localization and Pi efflux activity (Wege *et al*. 2016). The SPX-spanning region of PHO1 facilitates its interaction with PHO2, which is responsible for PHO1 degradation (Liu *et al*. 2012). The absence of the SPX domain in PHO1 does not affect its efflux activity in transient expression assay. However, it fails to restore the impaired root-to-shoot Pi translocation activity in *pho1* mutants, indicating that the SPX domain is crucial for PHO1’s functionality regulated by InsP/PP-InsP binding (Wege *et al*. 2016; Wild *et al*. 2016).

PHO2-mediated degradation of PHO1 occurs in endomembranes and is involved in vacuole proteolysis mediated by multi-vesicular bodies (Liu *et al*. 2012). Similarly, rice OsPHO2 also interacts with OsPHO1 to direct its degradation via a multi-vesicular body–mediated pathway (Wang *et al*. 2020), suggesting the conservation of the PHO2-PHO1 regulatory module among plants. The expression of *PHO2* is negatively regulated by miR399 (Bari *et al*. 2006) and positively regulated by the HD–ZIP III transcription factor PHB, which is inhibited by SHORT-ROOT (SHR) (Xiao *et al*. 2022). Studies have also revealed that OsCK2, a protein kinase, can enhance the degradation of OsPHO2 through the phosphorylation of OsPHO2 by OsCK2α3 (Wang *et al*. 2020).

### SULTR-like phosphorus distribution transporter (SPDT)

SPDT was first shown to play a crucial role in regulating P allocation in rice (Yamaji *et al*. 2017). SPDT is a plasma-membrane-localized transporter that facilitates Pi transport and is expressed in the xylem region of both enlarged- and diffuse-vascular bundles of the nodes. Loss-of-function of SPDT in rice decreased P levels in grains but increased P levels in leaves. These findings indicate that SPDT functions as a switch in the rice node to allocate Pi to the grains preferentially. Likewise, barley HvSPDT, mainly expressed in the nodes, also plays an essential role in loading P into grains (Gu *et al*. 2022). In addition, the primary function of vascular cambium-localized Arabidopsis AtSPDT was shown to preferentially distribute Pi to the growing tissues (Ding *et al*. 2020).

### Phosphate translocators

Phosphate translocators (PTs) localized in the inner envelope membrane of plastids play a crucial role in establishing the metabolic connection between the plastid stroma and cytosol. Different from the Pi transporters mentioned above, these PT facilitate a precise counter-exchange of Pi with phosphorylated intermediates, including triose-phosphate/phosphate translocator, phosphoenolpyruvate/phosphate translocator, glucose-6-phosphate/phosphate translocator, and pentose xylulose-5-phosphate/phosphate translocator. They were reported to be involved in vegetative tissue and gametophyte development, redox signaling in leaves, and acclimation responses of photosynthesis (Młodzińska and Zboińska 2016; Fabiańska *et al*. 2019).

## PHR1 as a central transcriptional regulator of PSRs

Arabidopsis PHR1 or rice PHR2 encoding an MYB coiled-coil transcription factor is a central positive regulator of the PSRs. PHR1 was initially identified from a genetic screen in Arabidopsis harboring a reporter gene driven by the IPS1 promoter highly induced by P starvation (Rubio *et al*. 2001). *phr1* mutants displayed multiple impairments in PSRs, including altered root-to-shoot Pi allocation, reduced accumulation of anthocyanin and carbohydrate, altered lipid composition and lipid remodeling genes, and impaired induction of several P starvation–induced (PSi) genes under P starvation (Rubio *et al*. 2001; Nilsson *et al*. 2007; Pant *et al*. 2015). Arabidopsis PHR1 transcript and protein accumulation are weakly responsive to P starvation; however, its activity is regulated by interacting with SPX proteins coupled with the cellular P status (Rubio *et al*. 2001; Puga *et al*. 2014).

PHR1 forms homodimers or heterodimers with its close homolog PHR1-like 1 (PHL1) and binds to P1BS elements present in the promoter of many PSi genes (Rubio *et al*. 2001; Bustos *et al*. 2010; Jiang *et al*. 2019b). Arabidopsis PHR1 and partially redundant PHL1 regulate the majority of transcriptional responses to P starvation, and P1BS elements are enriched in the promoter of PSi genes but not in P starvation-repressed (PSr) genes (Bustos *et al*. 2010). In P-starved *phr1phl1* mutants, nearly 70% of PSi genes and 50% of PSr genes showed decreased and increased expression, respectively, suggesting a direct and indirect regulation of transcriptional activation and repression responses (Bustos *et al*. 2010).

The other 3 Arabidopsis PHR1 homologs, PHL2, PHL3, and PHL4, have also been found to participate in PSRs to varying extents. PHL4 has a redundant function with PHR1/PHL1 but a minimal effect (Wang *et al*. 2018). PHL2 and PHL3 exhibit a specific interaction, and their expression is induced by P starvation, unlike PHR1 and PHL1. The transcriptomic analysis further suggests that PHR1/PHL1 and PHL2/PHL3 regulate various aspects of PSRs as separate regulatory modules (Wang *et al*. 2022). PHR1/PHL1 regulates genes involved in the cellular response to P starvation, Pi transport, and galactolipid biosynthetic process, whereas PHL2/PHL3 regulates genes related to the cellular response to P starvation, translation, and ribosome biogenesis. In rice, Arabidopsis *PHR1* homolog *OsPHR2* plays a crucial role in Pi homeostasis, and overexpressing *OsPHR2* increases PSi gene expression and results in P toxicity (Zhou *et al*. 2008). PHR1 homologs participating in cellular Pi homeostasis have also been identified in various plant species (Wang *et al*. 2022), suggesting that PHR proteins have a universal and predominant role in regulating PSRs.

Despite not being as prevalent as PHR1, other transcription factors have been identified as regulators of PSRs to different extents. MYB62, as a negative regulator of PSRs, is involved in the interplay between PSRs and gibberellic acid responses (Devaiah *et al*. 2009), and WRKY75 is a positive regulator of Pi uptake and a subset of PSi genes (Devaiah *et al*. 2007a). As mentioned, WRKY6 is a repressor of *PHO1*. It interacts with 2 W-box motifs in the promoter of *PHO1* and is degraded by 26S proteasome to relieve the suppression upon P starvation (Chen *et al*. 2009). Basic Helix-Loop-Helix 32 and zinc finger of *Arabidopsis thaliana* 6 were also reported to have roles in regulating Pi content and root architecture (Chen *et al*. 2007; Devaiah *et al*. 2007b).

## Intracellular P sensing and signaling

### SPX proteins as intracellular P sensors

Proteins harboring a hydrophilic and poorly conserved SPX domain have been identified in many eukaryotes, such as yeasts, plants, and mammals (Secco *et al*. 2012). Many yeast SPX domain–containing proteins are involved in Pi homeostasis, including Pi transporters Pho87, Pho90, and Pho91; polyphosphate synthase subunits of vacuole transporter chaperone VTC2-5; the cyclin-dependent kinase inhibitor Pho81; and the glycerophosphocholine phosphodiesterase 1 (Secco *et al*. 2012; Desfougères *et al*. 2016). Yeast Pho87 and Pho90 are plasma membrane–localized low-affinity Pi influx transporters, and their SPX domains act as auto-inhibitory domains to regulate Pi import (Hürlimann *et al*. 2009). The SPX domain of vacuolar Pi exporter Pho91 also regulates its activity through binding with PP-InsP (Potapenko *et al*. 2018). Additionally, PP-InsP-dependent binding on the SPX domains of yeast VTC complex is required for its polyphosphate polymerase activity (Gerasimaite *et al*. 2017).

**Figure 4.**
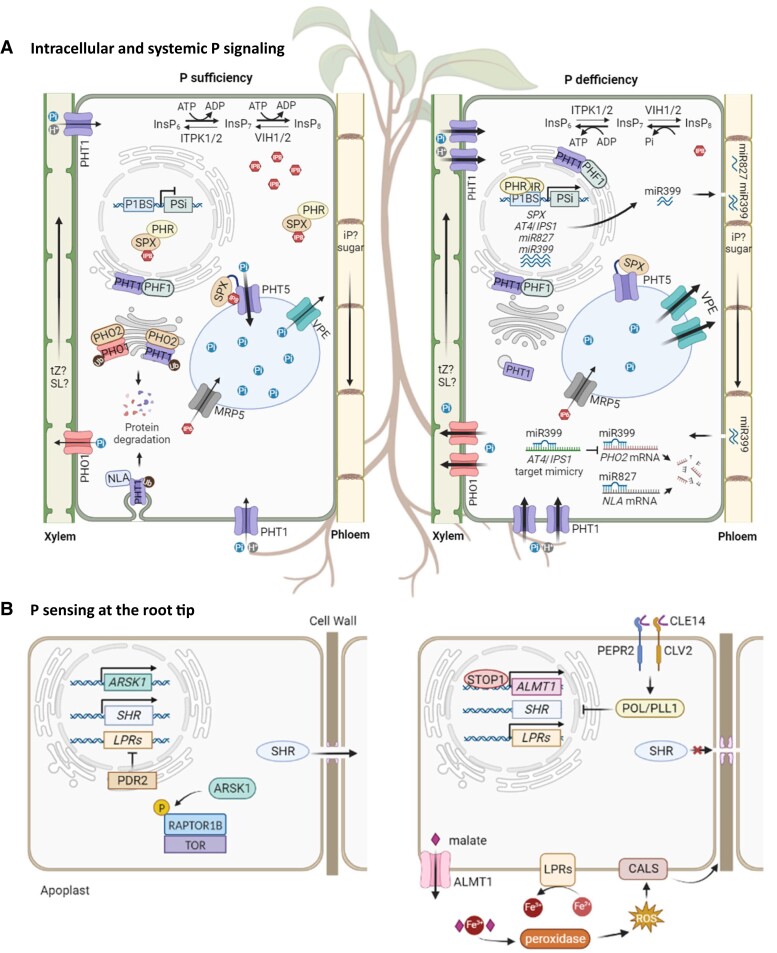
Intracellular and systemic signaling and local sensing to P availability at root tips. **A)** During P sufficiency, the synthesis of InsP_8_ is favored, which enables the interaction between SPX and PHR, suppressing PSi genes. Excess Pi in the cytosol is transported into the vacuole by PHT5. In the seeds, P is stored as InsP_6_ (IP6) in the vacuole by MRP5. PHO2 and NLA promote the degradation of Pi transporters PHT1 and PHO1 via ubiquitination (ub). Upon P deficiency, InsP_8_ (IP8) level is reduced. PHR is released and binds to the P1BS element as a dimer to activate PSi genes. PHF1 facilitates the targeting of PHT1 Pi transporters to plasma membranes. miR399 and miR827 repress *PHO2* and *NLA*, respectively, to increase the abundance of PHT1 and PHO1. *AT4*/*IPS1* transcripts can sequester miR399 to antagonize the effects of miR399. As indicated, miR399, miR827, sugar, strigolactone (SL), and cytokinin (tZ and iP) are potential systemic signals traveling via vascular tissues. **B)** Under P-sufficient conditions, ARSK1 is induced to phosphorylate RAPTOR1B, stabilizing the TOR1 complex and promoting root growth. Under P-deficient conditions, STOP1-ALMT1 modulates LPR1 activity, which controls Fe distribution in the root apical meristem, blocking SHR cell-to-cell movement by callose deposition. Meanwhile, the reception of CLE14 peptide activates POL/PLL1 phosphatases to inhibit root cell proliferation at transcript levels. Abbreviations: CALS, callose synthase; ROS, reactive oxygen species. Created with BioRender.com.

Arabidopsis SPX1 and SPX2 are nuclear proteins and function to repress PHR1 activity through physical interaction in the nucleus to sequester PHR1 from binding to the P1BS element of PSi genes (Fig. 4A) (Duan *et al*. 2008; Puga *et al*. 2014). Notably, the interaction between PHR1 and SPX1/2 highly depends on the cellular P status, with a strong interaction under P-sufficient conditions. Intriguingly, *SPX1/2* transcription is induced by PHR1 during P starvation, revealing a negative feedback regulatory loop between SPX1/2 and PHR1 (Puga *et al*. 2014). This mechanism is conserved in rice; OsSPX1/2 interacts with OsPHR2 to suppress the binding of OsPHR2 to the P1BS element (Zhou *et al*. 2008; Wang *et al*. 2014b). Other than in the nucleus, Arabidopsis SPX4 and rice OsSPX4 and OsSPX6 interact with PHR in the cytoplasm to restrain the nuclear translocation of PHR when P is replete (Lv *et al*. 2014; Zhong *et al*. 2018). Upon P deficiency, OsSPX4 and OsSPX6 undergo ubiquitin-mediated proteasomal degradation, thus releasing OsPHR2.

### InsP_8_ as a metabolic messenger for intracellular P signaling

Pi was initially considered to be a signaling molecule because of the attenuation of PSRs by phosphite (Phi, a nonmetabolized homolog of Pi) (Ticconi *et al*. 2001; Varadarajan *et al*. 2002). However, this concept was revised when the InsP/PP-InsP binding site of the SPX domain was revealed after solving the structure (Wild *et al*. 2016). The conserved amino acid residues defined as Pi binding cluster and lysine surface cluster together in the α-helix 2 and 4 of SPX domains form a positively charged surface, with higher affinities for InsP_6_ and 5-InsP_7_ than Pi. Moreover, the binding of InsP_6_ and 5-InsP_7_ but not Pi to OsSPX4 can facilitate the interaction with OsPHR2, with 5-InsP_7_ showing a higher affinity than InsP_6_ (Wild *et al*. 2016), supporting the notion that PP-InsP rather than Pi is the signaling molecule representing intracellular P status.

In Arabidopsis, the synthesis and hydrolysis of 5-InsP_7_ and 1,5-InsP_8_ are catalyzed by 2 bifunctional enzymes, inositol 1,3,4-trisphosphate 5-/6-kinase ITPK1/2 and diphosphoinositol pentakisphosphate kinase VIH1/2, respectively (Desai *et al*. 2014; Laha *et al*. 2015, 2019; Zhu *et al*. 2019; Whitfield *et al*. 2020; Riemer *et al*. 2021). The activity of ITPK1/2 and VIH1/2 can respond to changes in cellular ATP and Pi levels: high ATP favors kinase activity to synthesize 1,5-InsP_8_, as ATP levels decrease in response to P starvation, and ADP phosphotransferase activity of ITPK1 and phosphatase activity of VIH1/2 are stimulated to catalyze the hydrolysis of 5-InsP_7_ and 1,5-InsP_8_ (Laha *et al*. 2019; Zhu *et al*. 2019; Whitfield *et al*. 2020; Riemer *et al*. 2021). Inhibition of VIH1/2 phosphatase activity by Phi may explain the early observations of the Phi-mediated suppression of PSRs. Arabidopsis *vih1vih2* mutants displaying severe growth retardation exhibit an undetectable level of InsP_8_ but overaccumulation of Pi and constitutive expression of PSi genes when grown under P-sufficient conditions, implying that InsP_8_ is critical for plants to sense cellular P status (Dong *et al*. 2019b; Zhu *et al*. 2019). Furthermore, whereas the levels of InsP_6_ and InsP_7_ were relatively unchanged in response to P starvation, the level of InsP_8_ was significantly decreased (Kuo *et al*. 2018; Dong *et al*. 2019a). 1,5-InsP_8_ but not 5-InsP_7_ can restore the interaction between SPX1 and PHR1 in the Pi-depleted cell lysate (Dong *et al*. 2019b). These findings indicate that InsP_8_ is a signaling molecule communicating the cellular P level to control cellular Pi homeostasis through the SPX domain. A recent yeast study also revealed that 1,5-InsP_8_ specifically inactivates Pho81 through its SPX domain and that the reduction of 1,5-InsP_8_ upon P starvation triggers the yeast PHO pathway (Chabert *et al*. 2023). The authors further proposed 1,5-InsP_8_ as a universal signaling molecule in P sensing and signaling pathways among fungi, plants, and mammals (Chabert *et al*. 2023). It is worth noting that defective vacuolar storage of InsP_6_, as seen in *mrp5* mutants, results in elevated InsP_7_ and InsP_8_ levels (Desai *et al*. 2014; Riemer *et al*. 2021), suggesting InsP_6_ compartmentation may also play a role in P signaling.

Recent studies have investigated the structure-functional relationship of the SPX-PP-InsP-PHR complex. InsP_8_ promotes the binding between the InsP_8_-SPX1 complex and the coiled-coil domain of PHR1 (Ried *et al*. 2021). In the presence of InsP_6_ (used as a substitute for InsP_8_), OsSPX1 interacts with the MYB and the coiled-coil domains of OsPHR2 to form a complex with 1:1 stoichiometry, which disrupts the OsPHR2 dimer and further inhibits its binding to the P1BS element (Zhou *et al*. 2021). On the other hand, Guan *et al*. (2022) proposed that upon binding with InsP_6_, OsSPX2 forms a domain-swapped dimer and blocks the Myb and coiled-coil domains of OsPHR2 to abolish OsPHR2 dimerization and DNA binding; the SPX-InsP_6_-PHR complex was described as a 2:2:2 complex. This discrepancy might be due to the difference in conformational changes between different SPX proteins (Guan *et al*. 2022).

### P sensing and signaling at the root tips

Root tips are critical in sensing environmental P status and regulating root meristem activities in response to external stimuli. P starvation causes premature differentiation in the root meristem, inhibiting primary root growth (Sanchez-Calderon *et al*. 2005). The increase of cell wall stiffness in the root transition zone can be observed 2 hours after plants transfer to P-deficient conditions. This results in the restriction of root cell extension and inhibition of root growth (Balzergue *et al*. 2017). LPR1 and LPR2, 2 multicopper oxidases with ferroxidase activity, which function with PDR2, a P5-type ATPase, are involved in the control of iron (Fe) distribution in the root apical meristem (RAM) (Reymond *et al*. 2006; Svistoonoff *et al*. 2007; Ticconi *et al*. 2009; Müller *et al*. 2015; Naumann *et al*. 2022). Intriguingly, Fe distribution in P-depleted roots overlays with the callose deposition pattern in the cell wall, restricting the movement of SHR for stem cell specification (Müller *et al*. 2015). Loss of LPR significantly reduced class III peroxidase activity, which might affect ROS-mediated callose deposition, leading to cell wall softening (Müller *et al*. 2015; Balzergue *et al*. 2017). These studies demonstrate the role of the LPR1-PDR2 module in mediating Fe- and peroxidase-dependent P starvation–induced root meristem differentiation (Fig. 4B) (Balzergue *et al*. 2017; Mora-Macias *et al*. 2017). Later studies revealed the involvement of PDR2-mediated ER stress-dependent autophagy in controlling Pi starvation–induced root growth retardation (Naumann *et al*. 2019).

Organic acids such as malate and citric acid in root exudates are needed for solubilizing metal ion-conjugated Pi in soils (Ryan *et al*. 2001). The activation of ALMT1, a malate efflux channel, by SENSITIVE TO PROTON RHIZOTOXICITY (STOP1) is not only critical for aluminum tolerance (Liu *et al*. 2009; Sawaki *et al*. 2009) but also influences malate export in the apoplast of root tips and Fe distribution. The reduction of *LPR1* mRNA in *stop1* and *almt1* mutants and ineffective malate treatment on the long primary root phenotype in *lpr1* indicate that ALMT1-mediated malate-triggered responses promote LPR1-dependent root meristem differentiation (Fig. 4B) (Balzergue *et al*. 2017).

Arabidopsis CLAVATA3/ENDOSPERM SURROUNDING REGION 14 (CLE14) is a low-P–induced peptide signal found specifically in the cortex, endodermis, and stele of RAM, functioning downstream of LPR1/LPR2. Applying CLE14 peptides can trigger RAM differentiation even under P-sufficient conditions but does not affect callose deposition. Instead, CLE14 perceived by CLAVATA 2 (CLV2) and PEP1 RECEPTOR2 (PEPR2) activates 2 phosphatases, POLTERGEIST (POL) and POL-LIKE1, to repress *SHR* and *SCARECROW* at mRNA levels (Fig. 4B) (Gutierrez-Alanis *et al*. 2017); however, the underlying mechanism is still missing. Moreover, the crosstalk of these signaling pathways with phytohormone-mediated root growth regulation remains to be studied.

Although Fe distribution in root tips determines the root meristem activities, an Fe-independent regulatory pathway is suggested because the reduction of root growth was observed before changes in Fe accumulation in the short-term responses to P deficiency. Arabidopsis-root-specific kinase 1 (ARSK1), a receptor-like kinase, is expressed explicitly in P-sufficient roots and can phosphorylate regulatory-associated protein of TOR 1B (RAPTOR1B), a scaffold protein of the target of rapamycin complex 1 (TOR1) that integrates environmental cues to modulate cellular growth (Wu *et al*. 2019). Overexpressing ARSK1 or phosphorylated RAPTOR1B inhibited P deficiency–triggered primary root retardation, indicating the crucial role of ARSK1 in maintaining primary root growth under P-sufficient conditions via stabilizing TOR1 complex (Fig. 4B) (Cho *et al*. 2023). Therefore, PDR2-LPR1 and STOP1-ALMT1 modules are activated by low P and collectively regulate RAM activities after gradually reducing *ARSK1* levels.

### Systemic signaling

In addition to local sensing and signaling, optimizing adaptive responses to P starvation requires communication between roots and shoots via systemic signals traveling long distances in the vasculature. Several molecules are considered long-distance signals that regulate PSRs systemically (Fig. 4A).

miR399 and miR827 are evolutionarily conserved and induced explicitly by P starvation (Fujii *et al*. 2005; Hsieh *et al*. 2009; Lin *et al*. 2018). Their levels are highly increased in phloem sap during P starvation (Pant *et al*. 2008, 2009), suggesting their potential roles as P starvation signals moving from shoot to root to coordinate the shoot Pi demand and root Pi acquisition and translocation activity. miR399 and its target gene *PHO2* are coexpressed in vascular tissues (Fujii *et al*. 2005; Aung *et al*. 2006; Chiou *et al*. 2006). Suppression of *PHO2* by overexpression of miR399 results in overaccumulation of Pi in shoots due to increased uptake and root-to-shoot translocation of Pi (Chiou *et al*. 2006). The wild-type root grafted with miR399 overexpressing scion shows a high level of mature miR399, suppressing root *PHO2* transcripts and enhancing shoot Pi accumulation (Lin *et al*. 2008; Pant *et al*. 2008). Recently, the molecular basis underlying the miR399-mediated long-distance silencing was uncovered, by which miR399f/miR399f* duplex moves as a mobile entity and is unloaded in phloem pore pericycle in the root through plasmodesmata in a dose-dependent manner independent of its biogenesis, sequence context, and length (Chiang *et al*. 2023). Arabidopsis miR827 has also been reported to move from shoot to root (Huen *et al*. 2017). It is interesting to note that miR827 shifted its target preference during evolution; it targets *NLA* in Brassicaceae and Cleomaceae but targets PHT5 homologs in other species (Lin *et al*. 2018).

Plants accumulate sugars and starch in leaves upon P starvation (Nielsen *et al*. 1998; Nilsson *et al*. 2007), and the induction of PSi genes increased with increasing concentrations of exogenous sugars (Karthikeyan *et al*. 2007). Increased sucrose loading and shoot-to-root translocation enhanced the expression of PSi genes (Lei *et al*. 2011; Dasgupta *et al*. 2014); in contrast, impairment of sucrose translocation via stem girdling of white lupin suppressed the expression of PSi genes in Pi-starved roots (Liu *et al*. 2005). These findings support the systemic role of sugar in P signaling, but the underlying mechanism requires further investigation.

Phytohormones also play a role in P signaling. Strigolactones (SLs), a group of carotenoid-derived terpenoid lactones, inhibit shoot branching in plants (Gomez-Roldan *et al*. 2008; Umehara *et al*. 2008). It has been demonstrated that the reduced shoot branching under P starvation could be attributed to the increased levels of SL in xylem sap (Kohlen *et al*. 2011), indicating a systemic role for SLs in regulating shoot branching during P starvation to coordinate the shoot development with nutrient supply from roots. *N*^6^-(Δ^2^-isopentenyl) adenine (iP) and *trans-*zeatin (tZ) are primary forms of natural isoprenoid cytokinins in Arabidopsis with strong physiological activities (Sakakibara 2006). Early studies proposed cytokinins to be negative regulators of PSRs because the exogenous application of cytokinin suppresses the expression of PSi genes in Arabidopsis (Martín *et al*. 2000; Wang *et al*. 2006). iP and tZ are predominantly present in the phloem and xylem, respectively, suggesting cytokinins are able to function as systemic signals (Hirose *et al*. 2007). Silva-Navas *et al*. (2019) reported that even though *tZ* has a greater substantial repression effect on PSR genes, an increase in the cis-zeatin (cZ):tZ ratio in P-deprived plants to improve root architecture and sustain necessary cytokinin responses was observed. Nevertheless, the detailed systemic role of cytokinins in response to P starvation remains to be determined.

## Involvement of PSR factors in biotic interaction

Plants live with a diverse composition of microorganisms, and their relationship can be affected by plant internal nutrient levels or immune responses. It has been shown that plant P status significantly affects the interaction with beneficial and pathogenic microbes (Paries and Gutjahr 2023). Moreover, recent evidence reveals the involvement of PSR genes in regulating biotic relationships, providing profound insights into plant-microbe interaction.

### Regulation of arbuscular mycorrhizal (AM) symbiosis via SPX-PHR modules

**Figure 5.**
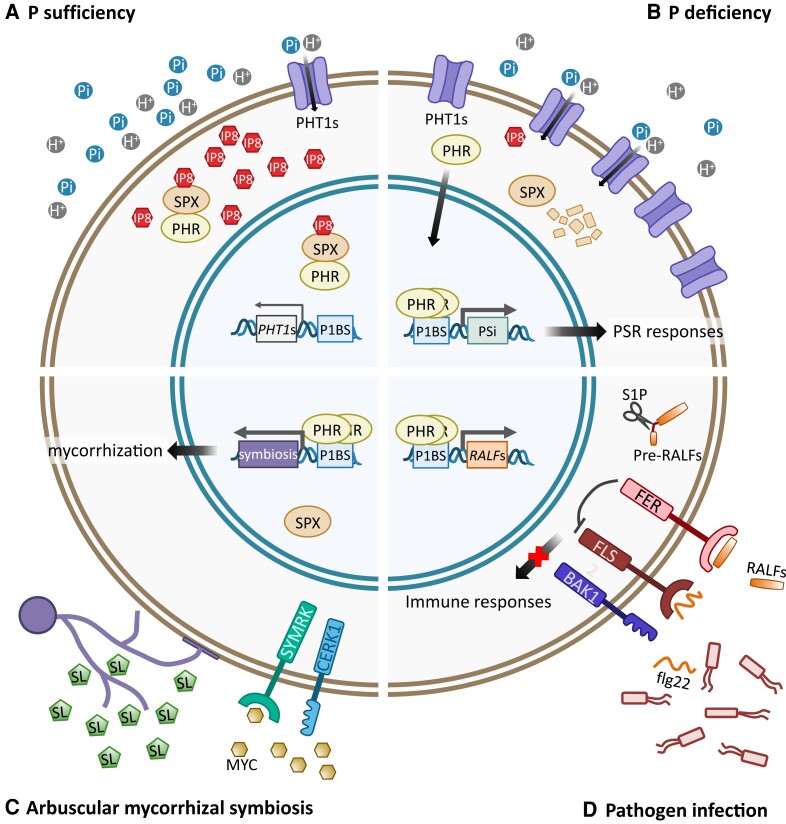
Versatility of SPX-PHR modules in response to P status and plant-microbe interaction. The SPX-PHR module not only regulates adaptive responses to P availability **A)** and **B)**, but it also regulates arbuscular mycorrhizal symbiosis **C)**, and the responses to pathogen infection **D)**, through controlling various sets of genes (see text for details). H in a circle represents a proton. Abbreviations: bbreviations: BAK1, BRI1-associated kinase 1; CERK1, Chitin Elicitor Receptor Kinase 1; flg22, flagellin peptide 22; FLS2, FLAGELLIN SENSING 2; MYC, Myc factor; S1P, site-one protease; SL, strigolactone; SYMRK, Symbiosis Receptor-like Kinase.

Due to the scarcity of Pi in soil, plants not only modulate Pi uptake efficiency but also form beneficial mutualistic relationships with microbes to enhance Pi acquisition (Fig. 5). Arbuscular mycorrhizal fungi (AMF) are soil-born microbes that can establish endophytic symbiosis with more than 80% of land plant species and provide mineral nutrients via highly branched structures, termed arbuscules, in root cortical cells in exchange for carbon source for survival (Chiu and Paszkowski 2019). Interestingly, P1BS elements are present in the promoter regions of several AM symbiosis-responsive genes, such as symbiosis-induced *PHT1* genes (Chen *et al*. 2011a; Lota *et al*. 2013). Rice *PHR2* is activated in arbuscule-containing cells (Shi *et al*. 2021), and overexpressing OsPHR2 induces symbiosis-responsive genes even without fungal infection (Shi *et al*. 2021; Das *et al*. 2022). Mutation at 3 rice PHR proteins severely impairs AMF colonization and arbuscular development. It alters the expression pattern of more than 60% symbiosis-responsive genes, including genes involved in pre-contact signaling, fungal entry, arbuscular development, and nutrient exchange (Shi *et al*. 2021; Das *et al*. 2022), demonstrating that PHR also functions in the center of AM symbiotic regulatory network (Fig. 5C).

As mentioned, modulation of PHR activity via the interaction with SPX proteins is widely validated in many plant species. Similarly, *Medicago SPX1* and *SPX3* are induced by low P, and SPX-PHR modules work in a P-dependent manner. In mycorrhizal roots, the activation of these 2 *SPX* genes is more confined to arbuscule-containing cells. Although the colonization efficiency in *spx1spx3* mutants is reduced, the enrichment of large arbuscules and the repression of arbuscular degeneration-related genes indicate that these 2 genes function redundantly in the control of arbuscular degradation (Wang *et al*. 2021). In addition, MtSPX1 and MtSPX3 control SL biosynthesis genes, the critical phytohormone in modulating shoot branching and AMF hyphae branching. The effects of MtSPX1 and MtSPX3 on the regulation of PSR and symbiosis were shown to be partially through the control of SL levels (Wang *et al*. 2021). In contrast, in rice *spx1/2/3/5* quadruple mutants, AMF colonization efficiency and arbuscular abundance are increased (Shi *et al*. 2021). In tomatoes, *Slspx1* mutants enhance AM colonization under Pi-replete conditions, whereas overexpression of *SlSPX1* inhibits the formation of AM symbiosis (Liao *et al*. 2022). Further characterization is required to determine whether the distinct effects of SPX protein are due to the difference of family members or the interacting partners in different species.

### Control of rhizobium-mediated nitrogen fixation via P status

Rhizobium-mediated nitrogen (N) fixation is critical for N supply and cycling in agriculture and ecosystems. In nodulating legumes, P supply to rhizobia is essential for maintaining nodule function and mutualistic relationship. Depletion of environmental P impairs nodulation and N fixation (Tang *et al*. 2001; Valverde *et al*. 2002). Soybean (*Glycine max*) PHT1;14, a high-affinity PHT1 family member, is induced by low P treatment, mainly in the junction area between roots and young nodules and in the nodule vascular bundles. It is one of the key players in transporting plant Pi to nodules (Qin *et al*. 2012). In addition to taking up Pi indirectly from host plants, Pi can be acquired directly by nodules from growth environments. Soybean PHT1;1 is upregulated in the plasma membrane of the outer cortex and the fixation zone of nodules in P-depleted roots. Overexpressing *GmPHT1;1* increases the fresh weight of nodules and the nitrogenase activity both under normal and P-deficient conditions, leading to the enhancement of N and P accumulation in plants and the final yield, supporting the significance of Pi transporters in Pi absorption by nodules and N fixation (Chen *et al*. 2019). *GmPHR1* and *GmPHR4* are expressed in the entire nodules and non-N-fixation region, respectively. Overexpressing *GmPHR1* enhances the expression of *GmPHT1;11* in nodules, leading to an increase in nodule Pi content and the size of the nodule, showing the importance of PHR-PHT1 module in nodules (Lu *et al*. 2020).

SPX proteins also regulate Pi homeostasis in nodules but not always via their association with PHR proteins. Soybean *SPX5* and *SPX8* are predominantly expressed in nodules and upregulated by P deficiency. Overexpressing either *GmSPX5* or *GmSPX8* increases the number and the fresh weight of nodules, resulting in higher N and P content in nodules than in the wild type (Zhuang *et al*. 2021; Xing *et al*. 2022). GmNF-YC4, a member of nuclear factor Y transcription factors, physically interacts with GmSPX5, and the association with GmSPX5 enhances the binding capability of GmNF-YC4 to the promoters of downstream target genes, such as asparagine synthetase-related genes. The phenotype of nodulation in overexpressing *GmNF-YC4* is largely overlaid with overexpressing *GmSPX5*, supporting the involvement of GmSPX5-GmNF-YC4 in nodule development and functions (Zhuang *et al*. 2021).

### Modulation of plant immunity against pathogens via P status

Excess P supply or Pi overaccumulation reduced the expression of defense-related genes, increasing the susceptibility to fungal pathogen *Magnaporthe oryzae* in rice (Campos-Soriano *et al*. 2020) and indicating the influence of external P in plant responses to pathogens. In contrast, P deficiency also suppresses plant immune response to promote the association with plant growth-promoting bacteria, which might facilitate Pi uptake efficiency or relieve the pressure of P scarcity (Tang *et al*. 2022). Thus, Pi uptake is tightly regulated to optimize the cellular P content for recruiting beneficial microbes. Among Arabidopsis PHT1 members, PHT1;4 is the major player in Pi uptake after recovering from P deficiency. Mutation at PHT1;4 reduces the susceptibility to the pathogen and upregulates pathogen elicitor-induced defense-related genes only under low P conditions (Dindas *et al*. 2022). Coincidently, overexpressing rice PT8 reduces the pathogen resistance (Dong *et al*. 2019b). These studies reveal the direct link between PHT1-dependent Pi uptake and immune responses.

The influences of PSR genes on the structure of root microbe communities also point out the connection between Pi signaling and immune responses. Results of transcriptomic and chromatin immunoprecipitation-sequencing analyses disclose that PHR1 regulates not only PSR genes but also a set of salicylic acid– and jasmonic acid–inducible genes, which are under the transcriptional control of PHR1 via the direct binding on the promoter regions (Castrillo *et al*. 2017). Moreover, the flagellin peptide flg22-responsive defense genes are upregulated in *phr1phl1* mutants. FERONIA (FER), a *Catharanthus roseus* receptor-like kinase 1-like (CrRLK1L) family member, and its peptide ligand, rapid alkalinization factor 23 (RALF23), can inhibit flg22-induced immune signaling (Stegmann *et al*. 2017). Interestingly, *RALF23* and several *RALF* genes are also the direct targets of PHR1 that are upregulated by low P treatment. Studies of overexpressing RALF23 or *fer* mutants support the involvement of the FER-RALF23 module in P starvation-mediated immune inhibition. All these observations together revealed that PHR1 has a negative role in regulating immune responses through the enhancement of the FER-RALK module (Fig. 5D) (Tang *et al*. 2022).

## Challenges and perspectives

Facing the limitation of Pi availability in nature, an increase in P fertilizer supply promotes crop yield, but plants acquire less than 30% of it (Vance *et al*. 2003). Tremendous progress has been made in establishing the regulatory network of Pi sensing and signaling over years of study, providing opportunities to accelerate high PUE crop development. Yet, many fundamental questions and gaps remain if such information is to be usefully translated to agriculture and ecological systems. For example, the underlying mechanisms of apoplastic responses to low external P to adjust the growth in root tips have been established; however, it is still unclear whether other plant cells can also sense extracellular P availability and how. Concerning intracellular sensing, the perception of InsP_8_ by SPX proteins determines the activation of PHR-mediated PSRs (Wild *et al*. 2016; Ried *et al*. 2021). Multiple enzymes are required to catalyze PP-InsPs synthesis, but understanding of the coordination of the enzyme activities in response to extra- or intracellular P levels remains limited. In addition, the direct link between external and internal P sensing is still missing.

Many studies have focused on increasing Pi acquisition to improve PUE. These include modifying root architecture to enlarge root surface area for Pi scavenging (Jia *et al*. 2018; Sun *et al*. 2018; Wang *et al*. 2019), enhancing the expression of PHTs to increase Pi acquisition and translocation (Song *et al*. 2014; Ouyang *et al*. 2016; Pontigo *et al*. 2023), modulating the secretion of root exudates involved in soil Pi solubilization (Pang *et al*. 2018; Robles-Aguilar *et al*. 2019; Wen *et al*. 2019) and promoting the interaction of plants with beneficial microbes to extend Pi source (Wen *et al*. 2019; Yahya *et al*. 2021).

Natural germplasms are valuable genetic resources for identifying novel alleles to improve the current cultivars' PUEs. Phenotypic and molecular characterization of various germplasm lines in crop species identified many quantitative trait loci associated with low P tolerance. Genome-wide association studies of soybean germplasms pinpointed a variant in the uORF of *GmPHF1* that contributes to P acquisition diversity (Guo *et al*. 2022b). Rice phosphate-starvation tolerance 1 (PSTOL1) is an enhancer of root growth and Pi uptake of aus-type cultivars grown in poor soils (Wissuwa *et al*. 2002; Gamuyao *et al*. 2012). Sequencing *PSTOL1* alleles in wild rice (*Oryza rufipogon*) grown in diverse environments found novel alleles associated with high Pi content, which might be used for breeding high PUE rice cultivars (Neelam *et al*. 2017). Ectopic expression of *OsPSTOL1* in wheat enhanced plant growth and root plasticity and promoted the induction of PHR-mediated P-starvation responses under low P conditions (Kettenburg *et al*. 2023). However, overexpressing wheat endogenous *TaPSTOL* homolog decreased PUE due to reduced yield but increased P accumulation in grains (Milner *et al*. 2018). Genome-wide association studies of soybean germplasm identified a variant in the uORF of soybean PHF1 as a key determinant for P acquisition efficiency in the field (Guo *et al*. 2022b). Nevertheless, most studies were conducted in greenhouses. Reproducing the traits in field conditions and integrating the knowledge on P sensing/uptake/recycling to improve plant PUE will be the main challenges for further application in breeding programs.

Coordinated acquisition and distribution of P and other nutrients are also required for optimizing plant growth and development. Thus, the interplay between P and other nutrients must be considered when modulating PUE via genetic strategies. Perception of nitrate strengthens the interaction of the nitrate sensor, OsNRT1;1B, with OsSPX4, resulting in OsSPX4 degradation, which leads to the activation of OsPHR2-mediated PSRs and NIN-like protein NLP3-mediated nitrate signalings (Hu *et al*. 2019). Meanwhile, Medici *et al*. (2019) identified PHO2 as another integrator of N signals to PSR pathways. These findings give insights into nitrate-regulated PSR responses, but little is known about the effects of ammonium and the role of P signaling in nitrate responses. Additionally, diverse functions of SPX and PHR homologs imply the different SPX-PHR modules present in vivo to exert transcriptional regulation. The underlying mechanism for controlling SPX-PHR formation and their roles in the interplay between P and other nutrient signaling requires further investigation.

As mentioned, Fe mediates P sensing in the root tips (Fig. 4B). Reciprocally, transcriptomic analysis also revealed the importance of P level in regulating Fe deficiency- or excess-responsive genes (Sanchez-Calderon *et al*. 2005; Thibaud *et al*. 2010). Rice HRZ, a Fe starvation-induced ubiquitin E3 ligase, mediates the protein degradation of OsPHR2. Conversely, OsPHR2 negatively regulates the *HRZ* transcript level to modulate Fe-deficient responses (Guo *et al*. 2022a), pointing out the regulatory loop in P-Fe interaction. Zn-dependent regulation of Pi accumulation has also been observed (Khan *et al*. 2014; Kisko *et al*. 2018), but knowledge about the underlying signaling and the coordination of P and Zn homeostasis is still lacking.

In terms of the role of plant P status in plant-microbe interaction, it is crucial to integrate PSR and immune responses for recruiting a specified set of microbes for survival. How to identify friends or foes and balance pathogen resistance and symbiotic interaction are fascinating topics about which little is known. Interestingly, PHR can directly activate genes required for AM symbiosis but repress genes involved in defense, suggesting that PHR appears to be a hub that coordinates immunity and symbiosis. However, the diversity and complexity of soil microbe composition challenge the integration of genetic studies and crop breeding programs. Large-scale studies and field trials are required to elucidate the interaction between P signaling and immune responses.

In nature, plants undergo multiple stresses. The rising temperature and CO_2_ levels aggravate the pressure and frequency of such stresses. However, the coordination between Pi signaling and other stress responses is rarely discussed. Recent research reported that overexpressing a chloroplast-localized Pi importer resulted in the overaccumulation of chloroplast Pi and phytic acids, leading to growth retardation under CO_2_-elevated conditions (Bouain *et al*. 2022). This result points out the importance of chloroplast Pi homeostasis in maintaining plant growth when plants face elevated CO_2_ levels. Despite being a challenging topic, more studies must be conducted to investigate the interplay between P and other stresses and underlying mechanisms, which need to be scrutinized in the field when applied to crop improvement.

## Dive Curated Terms

The following phenotypic, genotypic, and functional terms are of significance to the work described in this paper:

PHO1 Gramene: AT3G23430
PHO1 Araport: AT3G23430

PHO2 Gramene: AT2G33770
PHO2 Araport: AT2G33770

SHR Gramene: AT4G37650
SHR Araport: AT4G37650

WRKY6 Gramene: AT1G62300
WRKY6 Araport: AT1G62300

MYB62 Gramene: AT1G68320
MYB62 Araport: AT1G68320

PHB Gramene: AT2G34710
PHB Araport: AT2G34710

WRKY75 Gramene: AT5G13080
WRKY75 Araport: AT5G13080

IPS1 Gramene: AT3G09922
IPS1 Araport: AT3G09922

SPX3 Gramene: AT2G45130
SPX3 Araport: AT2G45130

PHR1 Gramene: AT4G28610
PHR1 Araport: AT4G28610

LPR1 Gramene: AT1G23010
LPR1 Araport: AT1G23010

PHF1 Gramene: Os07g09000
PHF1 Araport: Os07g09000

ALMT1 Gramene: AT1G08430
ALMT1 Araport: AT1G08430

LPR2 Gramene: AT1G71040
LPR2 Araport: AT1G71040

PDR2 Gramene: AT5G23630
PDR2 Araport: AT5G23630

PHR2 Gramene: Os07g25710
PHR2 Araport: Os07g25710

SPX1 Gramene: AT5G20150
SPX1 Araport: AT5G20150

SPX2 Gramene: AT2G26660
SPX2 Araport: AT2G26660

SPX4 Gramene: AT5G15330
SPX4 Araport: AT5G15330

STOP1 Gramene: AT1G34370
STOP1 Araport: AT1G34370

PHL2 Gramene: AT3G24120
PHL2 Araport: AT3G24120

PHL3 Gramene: AT4G13640
PHL3 Araport: AT4G13640

BAK1 Gramene: AT4G33430
BAK1 Araport: AT4G33430

CERK1 Gramene: AT3G21630
CERK1 Araport: AT3G21630

SEC12 Gramene: AT2G01470
SEC12 Araport: AT2G01470

At4 Gramene: AT5G03545
At4 Araport: AT5G03545

PHT5;1/VPT1 Gramene: AT1G63010
PHT5;1/VPT1 Araport: AT1G63010
